# Weekly self-monitoring and treatment adjustment benefit patients with partly controlled and uncontrolled asthma: an analysis of the SMASHING study

**DOI:** 10.1186/1465-9921-11-74

**Published:** 2010-06-10

**Authors:** Victor van der Meer, Henk F van Stel, Moira J Bakker, Albert C Roldaan, Willem JJ Assendelft, Peter J Sterk, Klaus F Rabe, Jacob K Sont

**Affiliations:** 1 Dept of Medical Decision Making, Leiden University Medical Center, Leiden, The Netherlands; 2 Dept of Public Health and Primary Care, Leiden University Medical Center, Leiden, The Netherlands; 3 Dept of Pulmonology, HAGA Hospital, The Hague, The Netherlands; 4 Dept of Respiratory Medicine, Academic Medical Center, University of Amsterdam, Amsterdam, The Netherlands; 5 Dept of Pulmonology, Leiden University Medical Center, Leiden, The Netherlands

## Abstract

**Background:**

Internet-based self-management has shown to improve asthma control and asthma related quality of life, but the improvements were only marginally clinically relevant for the group as a whole. We hypothesized that self-management guided by weekly monitoring of asthma control tailors pharmacological therapy to individual needs and improves asthma control for patients with partly controlled or uncontrolled asthma.

**Methods:**

In a 1-year randomised controlled trial involving 200 adults (18-50 years) with mild to moderate persistent asthma we evaluated the adherence with weekly monitoring and effect on asthma control and pharmacological treatment of a self-management algorithm based on the Asthma Control Questionnaire (ACQ). Participants were assigned either to the Internet group (n = 101) that monitored asthma control weekly with the ACQ on the Internet and adjusted treatment using a self-management algorithm supervised by an asthma nurse specialist or to the usual care group (UC) (n = 99). We analysed 3 subgroups: patients with well controlled (ACQ ≤ 0.75), partly controlled (0.75>ACQ ≤ 1.5) or uncontrolled (ACQ>1.5) asthma at baseline.

**Results:**

Overall monitoring adherence was 67% (95% CI, 60% to 74%). Improvements in ACQ score after 12 months were -0.14 (p = 0.23), -0.52 (p < 0.001) and -0.82 (p < 0.001) in the Internet group compared to usual care for patients with well, partly and uncontrolled asthma at baseline, respectively. Daily inhaled corticosteroid dose significantly increased in the Internet group compared to usual care in the first 3 months in patients with uncontrolled asthma (+278 μg, p = 0.001), but not in patients with well or partly controlled asthma. After one year there were no differences in daily inhaled corticosteroid use or long-acting β_2_-agonists between the Internet group and usual care.

**Conclusions:**

Weekly self-monitoring and subsequent treatment adjustment leads to improved asthma control in patients with partly and uncontrolled asthma at baseline and tailors asthma medication to individual patients' needs.

**Trial registration:**

Current Controlled Trials ISRCTN79864465

## Background

Recent international guidelines define asthma control in terms of two domains: impairment and risk [1,2]. The distinction between these two domains for assessing asthma control emphasizes the need to consider separately patients' functional capacity on an ongoing basis in the present and the risks for adverse events, such as side effects of medication, progressive lung function loss or exacerbations in the future.

Ongoing monitoring of asthma control (both impairment and risk) is required to determine whether the goals of therapy are met [1,3]. Well-validated self-assessment questionnaires are available to periodically monitor the level of asthma control [4-6]. Each of these instruments assesses the impairment domain by measuring asthma symptoms, limitation of activities and need for rescue medication. However, lung function is only included in the Asthma Control Questionnaire (ACQ) [4]. The periodic assessment of lung function is important, since it captures both asthma impairment at present and may detect future risk of progressive lung function and exacerbations [7,8].

The frequency of periodic monitoring depends on the phase of treatment [9]. At the initial phase intensive monitoring is required to evaluate the effect of treatment titration in order to achieve better asthma control. Once control has been achieved, the monitoring interval may be longer [9]. Monitoring frequency and subsequent treatment decisions therefore depend on the level of asthma control and vice versa.

We have conducted a trial in which the ACQ was used as a weekly monitoring tool and participants made treatment decisions according to an ACQ-based algorithm [10]. Asthma control and asthma related quality of life improved compared to usual physician-guided care, but the improvements were only marginally clinically relevant for the group as a whole [10]. In the present pre-planned analysis we investigated whether a simple index of asthma control can be used to predict the outcomes of Internet-based self-management. We hypothesized that self-management guided by weekly monitoring of asthma control tailors pharmacological therapy to individual needs and improves asthma control for patients with partly controlled or uncontrolled asthma.

## Methods

### Patients

Full details of the study methodology and subjects for the Self-Management of Asthma Supported by Hospitals, ICT, Nurses and General Practitioners (SMASHING) project at baseline have previously been published [10]. Briefly, the study enrolled 200 adults with asthma who were recruited from 37 general practices (69 general practitioners) in and around Leiden, The Netherlands, and from the outpatient department of Pulmonology of the Leiden University Medical Center. We included patients with physician diagnosed asthma, aged between 18 and 50 years who had a prescription of inhaled corticosteroids for at least three months in the previous year. We excluded patients on continuous oral glucocorticosteroid and patients on omalizumab. The study was approved by the Medical Ethics Committee of the Leiden University Medical Center. All participants gave written informed consent.

### Design

This analysis is part of a prospective, randomised controlled cost-effectiveness trial (ISRCTN79864465) [10]. Participants collected baseline data during a period of 2 weeks. They were trained to measure forced expiratory volume in 1 second (FEV_1_) daily with a hand-held electronic spirometer (PiKo1; Ferraris, UK) and were asked to report the highest value of three measurements in the morning on a designated Web application or by mobile phone text messaging. Along with the FEV_1 _value participants reported night time and daytime symptom scores. All participants were asked to complete the Asthma Control Questionnaire (ACQ) weekly on the Web application. During the baseline period participants received no feedback on lung function or clinical status.

After the baseline period, patients were randomised to either Internet-based self-management (Internet group) or usual physician-provided care (usual care group). The Internet group was instructed to use a personal Internet-based asthma action plan. This action plan required weekly completion of the ACQ via the Internet for a period of 1 year. After reporting the ACQ, participants instantly received a return message on the Website including advice on how to adjust treatment and a graphical representation of lung function and ACQ over time.

Patients in the usual care group did not use the ACQ throughout the study and did not receive weekly treatment advice. They received asthma care according to the Dutch general practice guidelines on adult asthma management, which recommend follow-up consultations every 2-4 weeks if asthma is not well controlled and medical review every year in well controlled asthma [11]. These national guidelines are based on international recommendations such as the GINA guidelines for asthma management and prevention [3].

After 3 months and after 1 year both the Internet and the usual care group collected asthma control data for a period of 2 weeks similar to the baseline period.

### Asthma Control Questionnaire

The ACQ is a 7-item questionnaire that has been validated to measure asthma control [4]. The items refer to asthma symptoms, rescue bronchodilator use and FEV_1_% of predicted normal. Responses are given on a 7-point scale and the overall score is the mean of the responses where 0 = totally controlled and 6 = severely uncontrolled.

### Asthma Therapy Assessment Questionnaire - control index

The ATAQ is a 20-item questionnaire that generates indicators of problems in asthma care. The control index of the ATAQ contains 4 items that refer to asthma symptoms, activity limitation and rescue bronchodilator use in the past 4 weeks. Sum scores range from 0 (no control problems) to 4 (control problems) [1,5].

### Treatment algorithm

Five pulmonologists, two general practitioners with special interest in respiratory disease and two respiratory epidemiologists participated in the development of the algorithm for the Internet-based asthma action plan. This algorithm was based on consecutive weekly ACQ scores. Two previous studies identified cut-off points for levels of asthma control. Juniper et al reported a cut-point of 0.75 for patients with well controlled asthma and a cut-point of 1.50 for patients with uncontrolled asthma [12]. Van den Nieuwenhof et al described cut-off points of 0.5, 1.0 and 1.5 to differentiate between the four severity levels of asthma in accordance with the GINA guidelines, although omitting the FEV_1_% of predicted normal [13].

**Figure 1:**
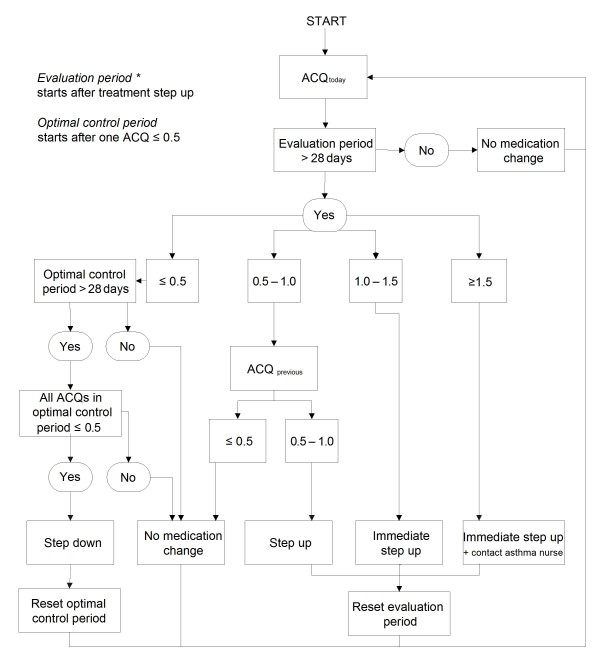
**Algorithm based on consecutive ACQ scores to adjust medical treatment **[10]. * At entry the evaluation period is bypassed.

Based on a clinically important difference of 0.5 the algorithm in our study uses three cut-points with 0.5 points differences: 0.5, 1.0 and 1.5 including the FEV_1_% of predicted normal [14]. It provides instructions to increase treatment (step-up) or decrease treatment (step-down) according to a pre-defined action plan. Figure 1 and table 1 show the treatment algorithm and action plan respectively. In brief, treatment step-up is advised when the ACQ score is above 1.0 once or between 0.5 and 1.0 twice consecutively and treatment step-down is advised after four weeks of ACQ scores below 0.5. When the ACQ score is above 1.5 the algorithm additionally advises to contact the asthma nurse or other health care provider. An evaluation period of four weeks without treatment changes follows after step-up instruction. Step-down instruction is followed by a period of four weeks (step-down period) in which no second step-down can be advised, but in case of deteriorating asthma, a treatment step-up is possible in this period.

**Table 1:** Treatment steps for the Internet-based asthma action plan [10]

Step*	Medication
1	As needed rapid-acting β_2_-agonist†
2	Low-dose inhaled glucocorticosteroids
3a	Low-dose inhaled glucocorticosteroids plus long-acting β_2_-agonist
3b	Medium-dose inhaled glucocorticosteroids
3c	High-dose inhaled glucocorticosteroids
4a	Medium-dose inhaled glucocorticosteroids plus long-acting β_2_-agonist
4b	High-dose inhaled glucocorticosteroids plus long-acting β_2_-agonist
4c	Contact asthma nurse‡: consider addition of leukotriene modifier
5	Contact asthma nurse‡: consider addition of oral glucocorticosteroid

**Figure 2:**
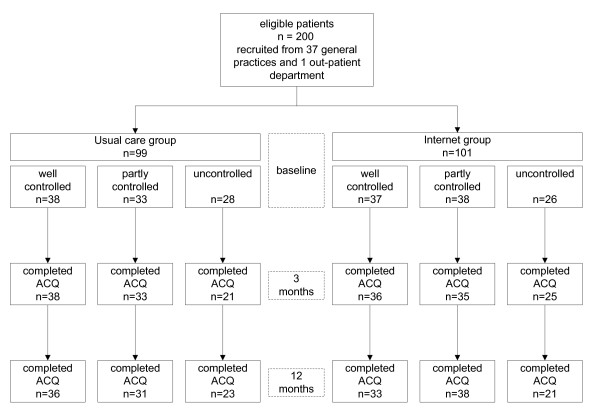
**Flow diagram of subject progress through the study**.

* Step numbers correspond with recommended steps in GINA guidelines figure 4.3-2. [3]

† Applies to all treatment steps

‡ Or other health care provider

### Monitoring adherence

Monitoring adherence was defined as the proportion of weekly completed Internet-based ACQs in the Internet group in each month of follow-up. We analyzed three subgroups of patients to allow evaluation of adherence for different levels of asthma control at baseline: well controlled (ACQ < 0.75), partly controlled (ACQ ≥ 0.75 to < 1.5) or uncontrolled asthma (ACQ ≥ 1.5) [1,12].

### Outcome measures

Asthma control was the primary process outcome. Asthma control was calculated as the average of ACQ scores during the two-week baseline and two-week end periods. The ATAQ control index measures the same construct as the ACQ (i.e. asthma control) and therefore acted as a measure of construct validity in order to support changes in ACQ.

Secondary outcome measures were the mean daily dose of inhaled corticosteroid (ICS), and the proportion of participants using long-acting β_2_-agonists (LABA) or leukotriene receptor antagonists (LTRA). Inhaled corticosteroid doses were reported as fluticasone equivalents. Data on pharmacological treatment were obtained from self-reports at baseline and after 3 months and 1 year.

We analyzed three subgroups of patients to allow evaluation of the treatment algorithm for different levels of asthma control at baseline: well controlled (ACQ < 0.75), partly controlled (ACQ ≥ 0.75 to < 1.5) or uncontrolled asthma (ACQ ≥ 1.5) [3,12].

### Sample size

With the 100 participants per study group and a standard deviation of changes in ACQ score of 0.69 we were able to detect at least a 0.28 difference between ACQ score changes in the two study groups (significance level 0.05 two-sided; power 0.80 one-sided) [15]. A clinically important decrease in ACQ score of at least 0.50 could thus be detected if at least 30 participants were present per subgroup [14].

### Statistical analysis

Differences in ACQ scores and inhaled corticosteroid doses between Internet and UC groups at two time points (3 and 12 months) were analyzed using multivariate linear regression modelling with a random intercept to adjust for repeated measures [16]. The construct validity of the ACQ as an outcome measure was evaluated by Pearson's correlation coefficients: 1) between ACQ and ATAQ control index at baseline and 12 months and 2) between change scores (12 months minus baseline value) of ACQ and ATAQ control index.

Differences in the proportion of patients using long-acting β_2_-agonists or leukotriene receptor antagonists between the two groups and at the two different time point were analyzed using multivariate population averaged logistic regression analysis with a random intercept [16]. Covariates in both regression models were baseline values of the appropriate outcome parameter, sex, age, education level, smoking status and type of care provider.

All analyses were carried out on an intention-to-treat basis. We used the statistical software package STATA 9.0 (StataCorp; College Station TX, US).

## Results

Figure 2 summarizes the participant flow during enrolment, allocation and follow-up [figure 2]. A total of 200 consented to participate in the randomised controlled study: 75 patients had well controlled asthma, 71 had partly controlled asthma and 54 had uncontrolled asthma at baseline [table 2]. Mean age was 36.3 and 31% were males. Smoking was reported more often in patients with uncontrolled asthma (33% current smokers) than in patients with partly controlled (8%) or well controlled asthma (3%). The ACQ at baseline was 0.43, 1.10 and 2.09 for the three groups, respectively. Inhaled corticosteroid use at baseline was 448, 483 and 620 μg/day, respectively. The use of long-acting β_2_-agonists was similar for the groups with partly and uncontrolled asthma (62% and 63%), and only slightly higher than in patients with well controlled asthma (55% long-acting β_2_-agonists use). Five patients used leukotriene receptor antagonists: one in the group with well controlled asthma, two in the group with partly controlled asthma and two in the groups with uncontrolled asthma.

**Table 2:** Baseline characteristics of 200 patients with mild to moderate persistent asthma who were randomised to Internet group or usual care group

	Well controlled asthma		Partly controlled asthma		Uncontrolled asthma	
	**Usual Care group** **(n = 38)**	**Internet group** **(n = 37)**	**Usual Care group** **(n = 33)**	**Internet group** **(n = 38)**	**Usual Care group** **(n = 28)**	**Internet group** **(n = 26)**

Age, mean yr (SD)	37.6 (7.5)	35.8 (8.9)	36.3 (10.1)	35.5 (9.7)	36.0 (6.9)	36.9 (7.6)
Male, no. (%)	12 (31.6)	11 (29.7)	10 (30.3)	8 (21.1)	7 (25.0)	13 (50.0)
Lower education, no. (%)	4 (10.5)	2 (5.4)	2 (6.1)	4 (10.5)	8 (28.6)	5 (19.2)
Current smoker, no. (%)	1 (2.6)	1 (2.7)	4 (12.1)	2 (5.3)	9 (32.1)	9 (34.6)
Subspecialty care, no. (%)	6 (15.8)	6 (16.2)	6 (18.2)	11 (29.0)	8 (28.6)	4 (15.4)

Duration of asthma, mean yr (SD)	16.8 (11.4)	15.5 (14.0)	20.4 (13.3)	16.1 (12.6)	15.8 (14.5)	14.2 (9.9)
Pre-bronchodilator FEV_1 _(% pred), mean (SD)	96.1 (11.4)	102.3 (13.4)	89.6 (13.6)	86.5 (9.6)	83.2 (14.9)	70.9 (15.9)
ACQ, mean (SD)	0.40 (0.23)	0.46 (0.18)	1.08 (0.22)	1.12 (0.23)	2.11 (0.55)	2.07 (0.44)
ATAQ control index, median (range)	1 (0-3)	1 (0-3)	1 (0-3)	1 (0-3)	2 (0-3)	2.5 (0-4)
Inhaled corticosteroids, mean μg/day (SD)	480 (368)	416 (236)	475 (377)	489 (309)	618 (311)	623 (316)
Long-acting β2-agonist, no. (%)	23 (60.5)	18 (48.7)	17 (51.5)	27 (71.1)	19 (67.9)	15 (57.7)
Leukotriene modifier, no. (%)	0 (0)	1 (2.7)	0 (0)	2 (5.3)	2 (7.1)	0 (0)

### Monitoring adherence

**Figure 3:**
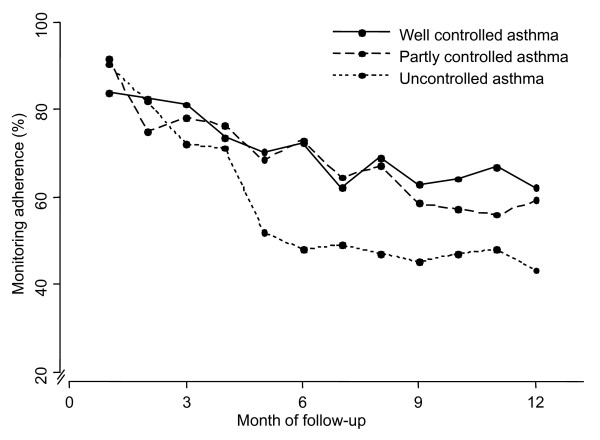
**Monitoring adherence (percentages) for patients with well controlled (n = 75), partly controlled (n = 71) or uncontrolled asthma at baseline (n = 54)**.

Overall monitoring adherence was 67% (95% CI, 60 to 74%). Adherence to ACQ monitoring gradually declined from the first month (88%) to the seventh month (60%) and then remained stable up to 1 year. Monitoring in the three subgroups was 71%, 68% and 58% during the one-year follow-up for well, partly and uncontrolled asthma at baseline, respectively [figure 3].

### Asthma control

There were no deviations from random allocation. ACQ scores at 12 months were provided by 69 (92%), 69 (97%) and 44 (81%) participants with well, partly and poorly controlled asthma, respectively.

**Figure 4:**
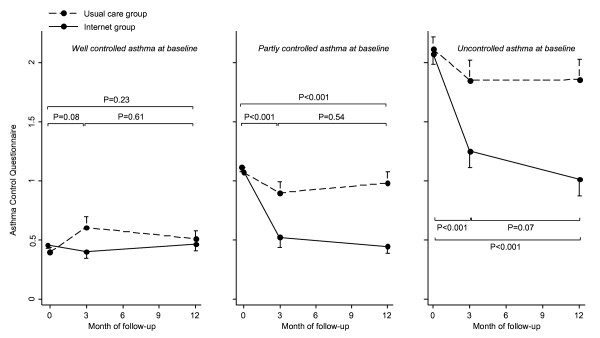
**ACQ scores during study follow-up for patients with well controlled (panel I; n = 75), partly controlled (panel II; n = 71) or uncontrolled asthma at baseline (panel III; n = 54)**. P-values represent statistical significance of change scores between Internet group and usual care. Error bars indicate the standard error of the mean.

Figure 4 shows the ACQ scores at baseline, 3 and 12 months of follow-up in the UC and Internet group for each baseline control level [figure 4]. In patients with well controlled asthma at baseline ACQ scores were not significantly different between the usual care and Internet group during follow-up. In patients with partly controlled asthma at baseline ACQ scores in the Internet group improved with -0.44 (95% CI, -0.67 to -0.22) and -0.51 (-0.73 to -0.29) after 3 and 12 months, respectively, compared to usual care. In patients with uncontrolled asthma at baseline ACQ scores in the Internet group improved with -0.57 (95% CI, -0.84 to -0.31) and -0.82 (-1.10 to -0.55) after 3 and 12 months, respectively, compared to usual care. Correlations between ACQ and ATAQ control index were 0.57 (p < 0.001) and 0.64 (p < 0.001) at baseline and 12 months, respectively. The correlation of change scores was 0.52 (p < 0.001).

### Pharmacological therapy

**Figure 5:**
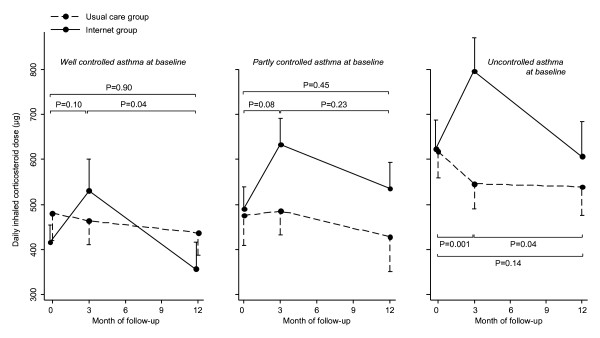
**Mean daily dose of inhaled corticosteroids (μg) during study follow-up for patients with well controlled (panel I; n = 75), partly controlled (panel II; n = 71) or uncontrolled asthma at baseline (panel III; n = 54)**. P-values represent statistical significance of change scores between Internet group and usual care. Error bars indicate the standard error of the mean.

Figure 5 shows the daily dose of inhaled corticosteroid (ICS) at baseline, 3 and 12 months of follow-up in the usual care and Internet group for each baseline control level [figure 5]. In patients with well controlled asthma at baseline the ICS dose increased non-significantly, followed by a significant decrease from 3 to 12 months (p = 0.042). At 12 months the ICS dose was similar for both groups: difference -9 μg (95% CI, -147 to 130). In patients with partly controlled asthma at baseline the ICS dose increased in the first 3 months and decreased in the next 9 months in the Internet compared to the usual care group, both changes being non-significant. At 12 months the ICS dose did not differ between the groups: difference 54 μg (95% CI, -86 to 194). Patients with uncontrolled asthma showed a significant increase in the first 3 months (278 μg, p = 0.001) followed by a significant decrease in the next 9 months (-149 μg, p = 0.043) in the Internet group compared to usual care. At 12 months the ICS dose was not significantly higher in the Internet group compared to usual care: difference 130 μg (95% CI, -43 to 303).

The number of patients using LABA or LTRA was not significantly different between the three baseline control levels and are therefore presented altogether. The proportion of patients using LABA was similar for the Internet and usual care group at 3 months (63% Internet and 62% usual care; p = 0.60) and 12 months (64% Internet and 58% usual care, p = 0.11), adjusted OR: 1.61 (95% CI, 0.74 to 3.48).

Only a few patients used LTRA. The proportion of patients using LTRA was significantly higher for the Internet group than usual care at 3 months (9% vs 2%, adjusted OR: 6.03 (95% CI, 1.03 to 35.4)), but not at 12 months (10% vs 4%, adjusted OR: 2.63 (95% CI, 0.67 to 10.3)).

## Discussion

This analysis provides insight into the effects of internet-based self-management guided by an electronic algorithm based on weekly assessment of asthma impairment on process outcomes for three different levels of asthma control at baseline. Adherence to the Internet-based monitoring instrument was 67%. The results show a considerable improvement in asthma control for patients with partly controlled or uncontrolled asthma at baseline without significant increases in inhaled corticosteroids, long-acting β_2_-agonists or leukotriene receptor antagonist use at 12 months.

This is the first randomised controlled evaluation of asthma self-management guided by a short validated questionnaire on asthma control. The current dynamic asthma management strategy reflects the varying and intermittent course of the disease, rather than doctor visits every three months as mentioned in international guidelines as they stand [1,3]. Our analysis reveals three important findings regarding asthma control, pharmacological therapy and monitoring adherence in the three subgroups of patients with different levels of asthma control at baseline.

First, the improvements in asthma control scores for patients with partly or uncontrolled asthma at baseline suggest a significant reduction of current asthma symptoms. Remarkably, control scores stabilised or even continued to improve after 3 months, while ICS doses decreased in patients with well or uncontrolled asthma at baseline. A possible explanation is that to achieve asthma control higher doses of anti-inflammatory therapy are needed than to maintain asthma control [3]. The reduced need for ICS may decrease future risk for side effects of medication.

Second, this asthma action plan is one of few that not only specifies action points to increase, but also to decrease treatment, which provides the possibility to tailor medication to individual needs. All three baseline control level groups showed a similar pattern of pharmacological therapy over time: an increase in inhaled corticosteroids in the first three months, followed by a decrease in the next 9 months. It can be seen that only for patients with uncontrolled asthma at baseline the inhaled corticosteroid dose significantly increased after three months. With regard to long-acting β_2_-agonists, the slight numeric difference of 6% in prescription can only partly explain the difference in findings in asthma control, since the magnitude of the improvements in asthma control suggests that the majority of the 59 patients with partly controlled or uncontrolled asthma experienced a clinically relevant improvement. Therefore, the patterns of increases and decreases in inhaled corticosteroids and long-acting β_2_-agonists reflected tailoring of medication to individual patients' needs rather than a mere increase of medication for the whole population.

Third, this study showed that weekly Internet-based monitoring is feasible in terms of monitoring adherence. In the groups with well and partly controlled asthma at baseline monitoring adherence of about 80% in the first 3 months decreased to 60% during the last months of follow-up. Despite declining monitoring adherence, asthma remained adequately controlled. This reflects the reduced need for monitoring once control of the disease has been achieved [9]. Patients with uncontrolled asthma at baseline monitored asthma control in 80% during the first 3 months (similar to patients with well and partly controlled asthma). However, In this group monitoring adherence declined to below 50% and asthma control did not reach the good control scores (below 0.75) as it did in the well and partly controlled groups. Efforts to optimise monitoring adherence may further increase asthma control.

Three methodological issues are of particular interest. The outcomes of our study were patient reported. Patient reported outcomes may have a risk of reduced validity compared to objective outcomes. Since the ACQ was both the target of the intervention and the main outcome measure, there was the possibility of a circular argument: a decrease in ACQ might have indicated that the algorithm worked, but not necessarily that asthma control improved. Therefore we used the ATAQ as a construct validity instrument and calculated correlations between ACQ and ATAQ. The moderate to good correlations, both cross-sectional and longitudinal, not only illustrated the effectiveness of a potent algorithm, but also supported our conclusion that indeed asthma control improved. With regard to medication reports, we asked patients to bring their inhalers at baseline and end visits, which enhanced the validity. However, patients may have reported different numbers of puffs than actually used or other types of inhalers than they actually brought to the visits.

Second, we recognize that the effect of the Internet-based self-management intervention can not solely be attributed to our treatment algorithm. We emphasize that, except for asthma monitoring and a medical treatment plan, a self-management asthma support programme should consist of asthma education, environmental control and medical review [17].

Third, our trial had a highly pragmatic attitude [18]. It was conducted in normal practice rather than an ideal research setting. Exclusion criteria were limited and the intervention was applied flexibly, i.e. patients' adherence to the monitoring and treatment algorithm was not strictly enforced, but patients and health care providers were allowed to make their own choices regarding monitoring and treatment as it would be in normal practice. The choice of this pragmatic design enhances applicability of the results in the real life.

## Conclusions

To conclude, weekly self-monitoring and subsequent treatment adjustment leads to improved asthma control in patients with partly and uncontrolled asthma at baseline and tailors asthma medication to individual patients' needs. Future asthma treatment strategies should incorporate continuous self-monitoring with use of a short validated questionnaire on asthma control.

## Competing interests

VM, HFS, MJB, ACR, WJJA, PJS, KFR and JKS have no declared conflict of interest.

## Authors' contributions

VM contributed to conception and design, acquisition of data and analysis and interpretation of data and drafted the manuscript. HFS contributed to analysis and interpretation of data and critically revised the manuscript. MJB contributed to acquisition of data and critically revised the manuscript. ACR contributed to analysis and interpretation of data and critically revised the manuscript. WJJA contributed to acquisition of data, analysis and interpretation of data and critically revised the manuscript. PJS contributed to conception and design, interpretation of data and critically revised the manuscript. KFR contributed to analysis and interpretation of data and critically revised the manuscript. JKS contributed to conception and design, acquisition of data and analysis and interpretation of data and critically revised the manuscript. All authors read and approved the final manuscript.
